# Extra-basal ganglia iron content and non-motor symptoms in drug-naïve, early Parkinson’s disease

**DOI:** 10.1007/s10072-021-05223-0

**Published:** 2021-04-16

**Authors:** Minkyeong Kim, Seulki Yoo, Doyeon Kim, Jin Whan Cho, Ji Sun Kim, Jong Hyun Ahn, Jun Kyu Mun, Inyoung Choi, Seung-Kyun Lee, Jinyoung Youn

**Affiliations:** 1 Department of Neurology, Gyeongsang National University Hospital, Jinju, Korea; 2 Center for Neuroscience Imaging Research, Institute for Basic Science (IBS), Suwon, Korea; 3 Department of Biomedical Engineering, Sungkyunkwan University, Suwon, Korea; 4 Department of Intelligent Precision Healthcare Convergence, Sungkyunkwan University, 2066 Seobu-ro, Jangan-gu, Suwon-si, Gyeonggi-do 16419 Korea; 5 Department of Biomedical Engineering, Gachon University, Incheon, Korea; 6 Department of Neurology, Samsung Medical Center, Sungkyunkwan University School of Medicine, Seoul, Korea; 7 Neuroscience Center, Samsung Medical Center, Irwon-ro 81, Gangnam-gu, Seoul, 135-710 Republic of Korea

**Keywords:** R2*, Quantitative susceptibility mapping, Iron, Parkinson’s disease, Non-motor

## Abstract

**Background:**

Although iron dyshomeostasis is associated with Parkinson’s disease (PD) pathogenesis, the relationship between iron deposition and non-motor involvement in PD is not fully understood. In this study, we investigated basal ganglia and extra-basal ganglia system iron contents and their correlation with non-motor symptoms in drug-naïve, early-stage PD patients.

**Methods:**

We enrolled 14 drug-naïve, early-stage PD patients and 12 age/sex-matched normal controls. All participants underwent brain magnetic resonance imaging to obtain the effective transverse relaxation rate (R2*) and quantitative susceptibility mapping (QSM). Deep brain structures, including the nucleus accumbens, caudate nucleus, putamen, globus pallidus, thalamus, hippocampus, and amygdala, were delineated using the FSL-FIRST; the substantia nigra, red nucleus, and dentate nucleus were segmented manually. Inter-group differences in R2* and QSM values, as well as their association with clinical parameters of PD, were investigated.

**Results:**

Substantia nigra and putamen R2* values were significantly higher in PD patients than in normal controls, despite no significant difference in QSM values. Regarding the non-motor symptom scales, PD sleep scale score negatively correlated with R2* values in the red nucleus and right amygdala, Scales for Outcomes in Parkinson’s disease-Autonomic scores were positively correlated with R2* values in the right amygdala and left hippocampus, and cardiovascular sub-score of Non-Motor Symptoms Scale for PD was positively associated with the QSM value in the left hippocampus.

**Conclusion:**

In this study, iron content in the extra-basal ganglia system was significantly correlated with non-motor symptoms, especially sleep problems and dysautonomia, even in early-stage PD.

**Supplementary Information:**

The online version contains supplementary material available at 10.1007/s10072-021-05223-0.

## Introduction

Iron plays a crucial biological role including mitochondrial respiration, myelin synthesis, and neurotransmitter production. Studies have shown an association between iron accumulation and many neurodegenerative diseases such as Parkinson’s disease (PD), Alzheimer’s disease (AD), multiple sclerosis, amyotrophic lateral sclerosis, and Huntington’s disease [1]. Iron dyshomeostasis was associated with oxidative stress and led to dopamine depletion, which could be implicated in PD [2]. In an AD mouse model, amyloid beta induced reduction of iron and iron oxidation state correlated with amyloid pathology [3]. Furthermore, neuroinflammation has recently been highlighted as an etiology of neurodegenerative diseases. As iron is present in oligodendrocytes, astrocytes, microglia, and neurons in the brain, it may provide a possible mechanism connecting neuroinflammation and degenerative diseases [4].

To date, various iron-sensitive magnetic resonance imaging (MRI) techniques such as susceptibility-weighted imaging, transverse relaxation rate (R2*), and quantitative susceptibility mapping (QSM) have been used to investigate the in vivo distribution of iron in the brain [5, 6]. In PD, iron accumulation in substantia nigra (SN) has been well established and many studies have been published on the relationship between iron concentration and disease progression [7, 8]. However, most previous studies of PD using iron-sensitive imaging focused on motor symptoms, whereas only a few studies have investigated non-motor symptoms [9–11]. Furthermore, these studies only focused on the cognition or total non-motor burden of PD. Additionally, most studies failed to eliminate the possible confounding effects of medication. Therefore, we sought to investigate the possibility that iron content in various brain regions may be associated with various non-motor symptoms (NMSs) in early-stage PD. Thus, in this study, the clinical association of iron content in drug-naïve, early-stage PD patients was investigated using R2* and QSM.

## Materials and methods

### Subjects

This study was approved by the Institutional Review Board of Samsung Medical Center, Seoul, Korea. Written informed consent was obtained from all enrolled participants. We recruited drug-naïve, early-stage PD patients and age/sex-matched normal controls at the movement disorder clinic, Samsung Medical Center, Seoul, Korea, from May to December 2017. The diagnosis of PD was based on the Movement Disorder Society PD diagnostic criteria [12], and early PD was defined as PD with less than 4 years of disease. All patients underwent N-(3-[^18^F]fluoropropyl)-2β-carbon ethoxy-3β-(4-iodophenyl) nortropane positron emission tomography in which we confirmed the typical pattern of presynaptic dopaminergic neuronal loss.

Parkinsonian motor symptoms were evaluated based on the Hoehn-Yahr (HY) stage [13] and Unified Parkinson’s Disease Rating Scale (UPDRS) part 3 total score [14] and four sub-scores: tremor, rigidity, bradykinesia, and axial symptoms [15]. For NMSs, the Korean Non-Motor Symptoms Scale for PD (K-NMSS), Beck’s anxiety inventory, Beck’s depression inventory, Innsbruck rapid eye movement sleep behavior disorder (RBD) inventory, Parkinson’s disease sleep scale (PDSS), Parkinson’s fatigue scale, Neuropsychiatric inventory, Scales for Outcomes in Parkinson’s disease-Autonomic (SCOPA-Aut), and Korean Mini-Mental Status Exam (K-MMSE) were used in this study [16–24].

Subjects were excluded if any of the following was detected: (1) contraindications for MRI scans, such as metallic implants or cosmetics; (2) significant motion during MRI acquisition; (3) structural brain lesions, including those due to territorial stroke, head trauma, or surgery; (4) dementia based on the K-MMSE score corrected with education year [24]; and (5) psychiatric disorders requiring medication or other medical conditions that could mimic PD, including atypical parkinsonism and musculoskeletal diseases.

### MRI acquisition and analysis

All enrolled subjects underwent brain MRI for R2* mapping and QSM using a 3T MRI scanner (Magnetom Prisma; Siemens Healthineers, Erlangen, Germany), in a session consisting of a localizer, T1-weighted imaging (magnetization-prepared rapid acquisition with gradient echo), T2-weighted imaging (turbo spin echo), and a multi-echo gradient echo (multi-echo GRE) scan. A typical scanning session lasted for approximately 25 min. The scan parameters for each scan are listed in Supplementary Material 1.

R2* for each voxel was calculated by a mono-exponential fit of the magnitude data from all echo-times after removing the Rician noise [25]. All image processing was performed in MATLAB (MathWorks, Natick, MA, USA). The R2* map acquisition process is depicted schematically in Supplementary Material 2. The complex data from the multi-echo GRE scans were also processed for QSM, using the publicly available software package STI Suite (https://people.eecs.berkeley.edu/~chunlei.liu/software.html). The QSM results were used to generate additional contrast to aid in the segmentation of certain deep brain nuclei (see below) [26] which normally exhibit poor T1-weighted contrast.

### Segmentation of brain structures

Apart from the basal ganglia structures that were mainly involved in PD, we also investigated the red nucleus (RN) and dentate nucleus (DN) that had cortical or striatal connections, which could influence PD motor and non-motor symptoms [27, 28]. We also assessed the limbic system including the amygdala, hippocampus, and thalamus, since it regulates various NMSs such as autonomic, emotional, and memory function.

**Fig. 1:**
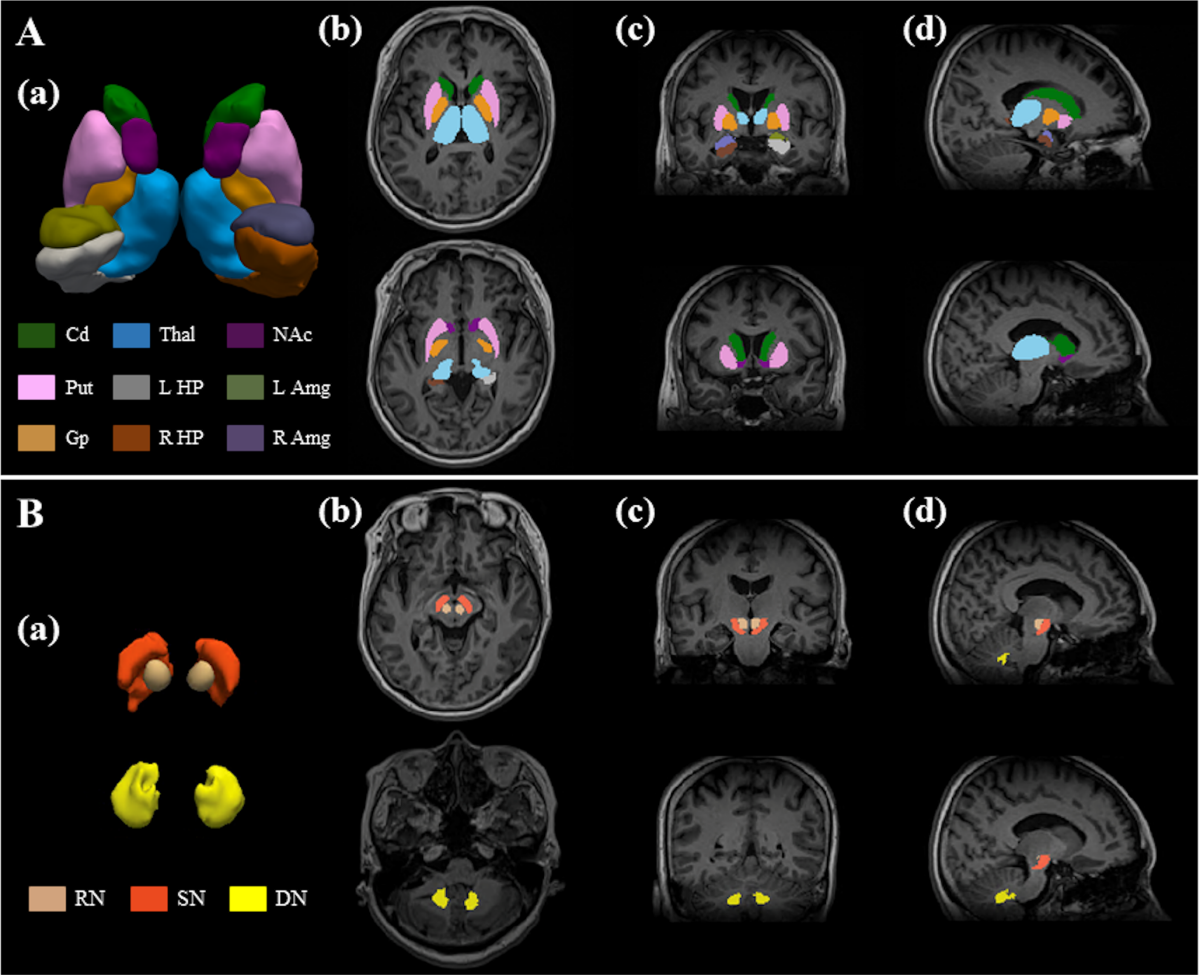
(**A**) Segmentation of the regions of interest (ROIs) in the deep brain structures of a representative volunteer by FSL. Illustrated are three-dimensional rendering of the ROI masks with automatic segmentation from FSL (a), and axial (b), coronal (c), and sagittal (d) plane views on the T1-weighted images. (**B**) Segmentation of the manually drawn ROIs of SN, RN, and DN. Abbreviations*.* Cd, caudate nucleus; Put, putamen; Gp, globus pallidus; Thal, thalamus; NAc, nucleus accumbens; R/L HP, right/left hippocampus; R/L Amg, right/left amygdala; SN, substantia nigra; RN, red nucleus; DN, dentate nucleus

The T1-weighted images were used to segment the bilateral nucleus accumbens, caudate nucleus, putamen, globus pallidus, hippocampus, amygdala, and thalamus in an automated workflow based on the FIRST function of the FSL (created by the Analysis Group, FMRIB, Oxford, UK) (Fig. 1A). The SN, RN, and DN were manually segmented by a neurologist (M.K.) based on QSM (Fig. 1B). This was repeated by the same person 6 months later, and the consistency between the two manual segmentations was verified based on intraclass correlation coefficients, which were over 0.86 for all areas (0.992, left SN; 0.869, right SN; 0.998, left RN; 0.999, right RN; 0.999, left DN; and 1.000, right DN).

### Statistical analysis

All data were presented as the mean and the standard deviation over the volunteers in each group. The independent t-test or Mann-Whitney *U* test was used to compare the baseline characteristics, R2*, and QSM values between the two groups. *p* values < 0.05 were considered significant. The correlations between the imaging parameters (R2* and QSM values) and clinical (motor and non-motor) scores were assessed using a Spearman rank correlation test wherein age and sex were controlled. When the correlation with K-MMSE score was investigated, age, sex, and education years were controlled. Bonferroni correction was used for multiple comparisons, with a significance level *α* = 0.05/45. For all statistical analyses, the commercially available software package IBM SPSS Statistics version 25 (SPSS Inc., Chicago, IL, USA) was utilized.

## Results

### Subjects and clinical characteristics

Overall, 14 drug-naïve PD patients and 12 normal controls were recruited for this study. Demographic and clinical data are presented in Table 1. Among the PD patients, the mean UPDRS part 3 score was 11.6 ± 8.5, and the mean HY stage was 1.9 ± 0.6. With regard to the NMSs, gastrointestinal symptoms, anxiety, and RBD, which are known as pre-motor symptoms in PD, were more prominent in PD patients compared to those in control group participants.

**Table 1:** Demographic and clinical data of enrolled subjects

		NC (*n* = 12)	PD (*n* = 14)	*p*-value
General information				
Age (years)		58.5 (9.7)	64.1 (9.3)	0.147
Sex (M:F)		5:7	9:5	0.431
Education year (years)		13.1 (4.8)	10.2 ( 5.5)	0.170
Motor symptoms				
Age of onset		NA	62.1 (9.4)	
Disease duration (years)		NA	2.0 (2.0)	
Dominance (Rt: Lt)		NA	10: 4	
UPDRS part 3	Tremor	NA	0.8 (0.7)	
	Bradykinesia	NA	4.0 (3.5)	
	Rigidity	NA	1.9 (2.7)	
	Axial symptom	NA	4.9 (3.7)	
	Total score	NA	11.6 (8.5)	
HY stage			1.9 (0.6)	
Non-motor symptoms				
NMSS	Cardiovascular	0.8 (1.4)	1.4 (3.7)	0.952
	Sleep/fatigue	2.6 (4.1)	4.9 (7.6)	0.123
	Mood/cognition	1.3 (1.4)	3.8 (6.6)	0.577
	Perceptual/ Hallucination	0.2 (0.4)	0.5 (1.3)	0.708
	Attention/memory	2.3 (4.1)	2.3 (2.5)	0.339
	Gastrointestinal	0.0	2.6 (3.7)	0.001*
	Urinary	0.7 (0.9)	1.9 (2.6)	0.194
	Sexual function	0.8 (1.2)	1.8 (4.8)	0.814
	Miscellaneous	1.1 (2.1)	2.8 (4.7)	0.168
	Total score	9.8 (12.5)	21.9 (27.4)	0.060
BAI		3.25 (4.9)	10.0 (11.7)	0.008*
BDI		3.8 (3.5)	6.9 (8.4)	0.339
RBD inventory		10.0 (0.9)	9.0 (1.4)	0.015*
PDSS		126 (33)	129.0 (29)	0.571
PFS		23.0 (17)	21.5 (27)	0.642
NPI		0.0 (0)	0.0 (4)	0.101
SCOPA-Aut		6.5 (5.8)	12.4 (8.8)	0.058
K-MMSE		28.2 (1.8)	27.8 (1.7)	0.586

Abbreviations. *PD*, Parkinson’s disease; *NC*, normal controls; *UPDRS*, Unified Parkinson’s Disease Rating Scale; *HY*, Hoehn and *Yahr*; *NMSS*, Non-Motor Symptoms Scale; *BAI*, Beck’s anxiety inventory; *BDI*, Becks depression inventory; *RBD*, rapid eye movement sleep behavior disorder; *PDSS*, Parkinson’s disease sleep scale; *PFS*, Parkinson’s fatigue scale; *NPI*, Neuropsychiatric inventory; *SCOPA-Aut*, Scales for Outcomes in Parkinson's disease-Autonomic; *K-MMSE*, Korean Mini Mental State Examination

*Statistically significant (*p* < 0.05)

The values are presented as mean (standard deviation). Age, education year, SCOPA-Aut, and K-MMSE were assessed using an independent *t*-test; for the rest of the items, the Mann-Whitney *U* test was used

### Comparison of iron accumulation between patients with PD and normal controls

There was no significant difference in both R2* relaxation rates and QSM values between the left and right structures for all recruited subjects. Therefore, we used bilaterally averaged R2* and QSM values, except those of the hippocampus and amygdala, where functional laterality has previously been reported [29]. When we compared these R2* and QSM values between PD patients and normal controls, the R2* values of the SN and putamen were significantly higher in PD patients than in the normal controls, but there was no significant difference in the QSM values between the two groups (Table 2).

**Table 2:** Results of R2* and QSM analyses in the PD and NC groups

Structures	NC (*n* = 12)	PD (*n* = 14)	*p*-value
R2* (1/s)			
Nucleus accumbens	20.7 (1.9)	22.5 (2.5)	0.057
Caudate	23.5 (2.3)	25.9 (3.2)	0.080
Putamen	27.8 (2.9)	31.2 (4.9)	0.031*
Globus pallidus	37.7 (5.2)	39.2 (4.5)	0.465
Thalamus	19.8 (0.9)	20.3 (1.2)	0.252
Substantia nigra	29.1 (2.8)	31.9 (3.5)	0.034*
Red nucleus	26.4 (2.8)	28.8 (3.3)	0.111
Dentate nucleus	30.0 (3.2)	31.4 (2.7)	0.239
R. amygdala	15.5 (1.1)	16.5 (1.8)	0.141
L. amygdala	16.6 (1.2)	16.5 (1.5)	0.804
R. hippocampus	17.7 (1.0)	17.7 (1.6)	0.977
L. hippocampus	17.2 (1.0)	17.4 (1.0)	0.740
*QSM (ppm)*			
Nucleus accumbens	0.0056 (0.0092)	0.0118 (0.0075)	0.073
Caudate	0.0278 (0.0045)	0.0298 (0.0041)	0.262
Putamen	0.0259 (0.0073)	0.0303 (0.0086)	0.178
Globus pallidus	0.0549 (0.0151)	0.0516 (0.0128)	0.550
Thalamus	0.0001 (0.0027)	− 0.0005 (0.0026)	0.561
Substantia nigra	0.0703 (0.0106)	0.0716 (0.0151)	0.806
Red nucleus	0.0712 (0.0123)	0.0677 (0.0105)	0.434
Dentate nucleus	0.0502 (0.0158)	0.0396 (0.0104)	0.052
R. amygdala	0.0009 (0.0095)	0.0006 (0.0079)	0.898
L. amygdala	− 0.0020 (0.0079)	0.0007 (0.0082)	0.403
R. hippocampus	0.0017 (0.0045)	0.0019 (0.0032)	0.913
L. hippocampus	0.0009 (0.0020)	0.0015 (0.0044)	0.667

Abbreviations. *PD*, Parkinson’s disease; *NC*, normal controls; *R.*, right; *L.*, left

*Statistically significant (*p* < 0.05)

Values are given as mean (standard deviation). Caudate nucleus, putamen, and red nucleus were assessed using the Mann-Whitney *U* test; for the rest of the items, the independent *t*-test was used

### Correlation analysis of iron content with motor and NMSs in patients with PD

**Fig. 2:**
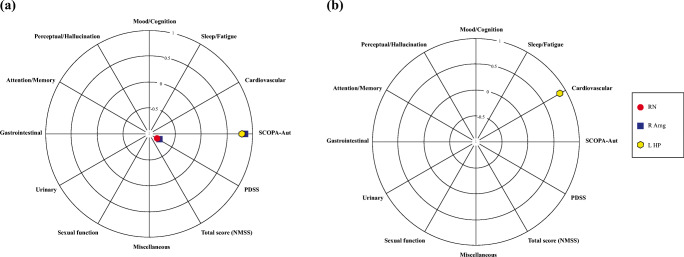
Correlation analysis results between R2* (**a**) QSM values (**b**) and non-motor symptoms. Only statistically significant correlation values are shown

In terms of motor symptoms, there was no significant correlation between both the R2* and QSM values and the severity of motor symptoms assessed using the UPDRS part 3 total score and sub-scores and HY stage regardless of age and sex. However, the iron contents of several extra-basal ganglia structures correlated with various NMSs when age and sex were controlled (Fig. 2). PDSS score was negatively correlated with the R2* values in the RN (*r* = − 0.791, *p* = 0.024) and the right amygdala (*r* = − 0.758, *p* = 0.048), and SCOPA-Aut values were positively correlated with R2* in the right amygdala (*r* = 0.789, *p* = 0.024) and the left hippocampus (*r* = 0.756, *p* = 0.048). In terms of QSM, the NMSS cardiovascular sub-score was positively associated with the QSM value in the left hippocampus (*r* = 0.760, *r* = 0.048).

## Discussion

To our knowledge, this is the first study to investigate the relationship between iron deposition and diverse NMSs in drug-naïve PD patients. Iron accumulation is known to be associated with PD pathogenesis, but whether it is a cause or a consequence of neurodegeneration has not yet been elucidated. We performed an explorative study to investigate the correlation between iron content and clinical assessment, focusing especially on NMSs in drug-naïve, early-stage PD patients using R2* and QSM. Intriguingly, we found significant correlations between the R2* and QSM values of various deep brain structures, especially those of the extra-basal ganglia system, and NMSs of PD, while there was no significant correlation with motor symptoms.

In accordance with our results, increased iron content in the SN of PD patients has been observed in postmortem as well as in vivo radiologic studies [5, 6]. Regarding iron distribution outside the SN, the results have been less consistent. PD patients were found to have increased iron content in the globus pallidus, putamen, and SN in a previous study [9], while reduced putaminal iron levels were reported in another study [30].

The correlation between iron content and motor symptoms was investigated in previous studies, but the results were also inconsistent. For example, UPDRS part 3 scores showed no correlation with iron content in some studies [31–33], while other studies demonstrated a significant correlation [7, 9]. In the present study, we did not observe any significant correlation between iron deposition and motor symptoms, although R2* values were significantly increased in the SN and putamen in PD patients than in normal controls. Iron homeostasis is disrupted in the early phase of neurodegeneration, and iron accumulation may already have started even before mild Parkinsonism appears, resulting in a lack of correlation of R2*and QSM with motor symptoms. Similarly, one previous study showed that SN iron content measured using R2* and QSM correlated with changes in UPDRS part 1 and part 3 scores, respectively, only in the late stage of PD [8]. PD is a wide-spectrum disorder with various clinical presentations; thus, more studies with larger samples of homogenous PD patients are needed for consensus on this issue.

In terms of NMSs, only a few studies have demonstrated an association between iron content and non-motor involvements in PD. Studies have shown that magnetic susceptibility values do not differ between PD patients with high and low NMS burdens [11] and that the severity of NMSs, as assessed based on the UPDRS part 1 score, does not correlate with magnetic susceptibility in the SN pars compacta [31]. However, susceptibility-weighted imaging showed that iron deposition in the SN was correlated with cognitive impairment, sleep disturbance, and autonomic dysfunction in PD patients [34]. Besides the iron content in SN, cognitive impairment was shown to be associated with cerebral iron burden when assessed using QSM [9, 10]. These results suggest that iron accumulation is not exclusive to the SN but can occur in extra-basal ganglia regions and could be associated with PD NMSs.

In this study, we found significant correlations between extra-basal ganglia structure R2* and QSM values and autonomic dysfunction and sleep problems, and our results are in line with those of previous studies—early-stage PD patients exhibited significant loss of right amygdala gray matter density, which was correlated with the SCOPA-Aut score [35]; more severe alpha-synuclein and tau pathologies were observed in the amygdala of PD patients with sleep disturbances [36]; and blood pressure variability was associated with hippocampal volume [37]. Lastly, the RN has been suggested to have a compensatory role in PD progression [28] and is connected to cortical and subcortical regions, including the hypothalamus, that regulates wakefulness [27]. Thus, the RN R2* value in early-stage PD may be associated with sleep problems.

Although both R2* and QSM were calculated from the same, multi-echo GRE scans, we observed a discrepancy between R2* and QSM in terms of their statistically significant discrimination between PD and control groups, as well as their correlation with clinical symptoms. Such discrepancy can be attributed to several factors. First, as paramagnetic (such as due to iron) and diamagnetic (such as due to myelination) tissue properties have different effects on R2* and QSM [38], variable tissue magnetic properties arising from early PD pathologic changes may have been differentially reflected in R2* and QSM. Second, QSM is known to be affected by the subjects’ head orientation with respect to the main magnetic field. In a recent study [39], such dependence was reported to be about 0.01 ppm per 5° in the deep brain region. Since the head orientation was not controlled in our study, such orientation dependence could have added to the overall variability of our QSM results (Supplementary Material 3). Lastly, while R2* was calculated with a relatively robust method of voxel-wise fitting of the magnitude data, QSM was primarily based on the image phase, which is more prone to errors due to physiological motion and streaking artifacts [38, 39]. Along with the small sample size, such increased variability may have rendered QSM weak in its statistical power to distinguish between different subject groups. This is in accordance with a previous study that reported low statistical significance in a small-sample QSM analysis, conducted with focus on the SN of PD patients [40].

Our study has certain inherent limitations. The first limitation was that the sample size was small. However, we formulated a homogeneous and qualified clinical cohort for this study. All patients fulfilled the diagnostic criteria of PD [12] and were free of confounding effects of medication and variable disease severity. Further studies with larger samples of homogenous PD patients are needed to draw more robust conclusions. Second, although education year-corrected K-MMSE score exhibited excellent discriminative power for dementia [24], K-MMSE may not be a sensitive tool to detect mild cognitive decline in early-stage PD. Full neuropsychological tests may allow to identify the presence of mild cognitive decline and to reveal its association with R2* and QSM values in future studies. Additionally, the PD patients were older than the normal controls, although the difference was not statistically significant; thus, age-related changes in brain iron levels were not reflected in our analysis, potentially leading to a bias in the results. Nevertheless, the strength of this study is that various non-motor symptom scales were used to evaluate drug-naïve, early-stage PD patients with a focus on the association between iron content and NMSs.

In conclusion, increased iron levels were observed in the SN and putamen of early-stage PD patients who were not on any medication. The iron levels in the limbic system and RN were especially found to correlate with sleep problems and dysautonomia even in early-stage PD. Our study may provide insight into the relationship between iron deposition and NMSs in PD. A future study that investigates the role of extra basal ganglia iron in PD NMSs is warranted.

## Supplementary Information

ESM 1 (DOCX 142 kb)

## Data Availability

The datasets generated during and/or analyzed during the current study are not publicly available because patient information is included. However, they are available from the corresponding authors on reasonable request.

## References

[1] Ward RJ, Zucca FA, Duyn JH, Crichton RR, Zecca L (2014). The role of iron in brain ageing and neurodegenerative disorders. Lancet Neurol.

[2] Surendran S, Rajasankar S (2010). Parkinson’s disease: oxidative stress and therapeutic approaches. Neurol Sci.

[3] Telling ND, Everett J, Collingwood JF, Dobson J, van der Laan G, Gallagher JJ, Wang J, Hitchcock AP (2017). Iron biochemistry is correlated with amyloid plaque morphology in an established mouse model of Alzheimer's disease. Cell Chem Biol.

[4] Ong WY, Farooqui AA (2005). Iron, neuroinflammation, and Alzheimer’s disease. J Alzheimers Dis.

[5] Wang J-Y, Zhuang Q-Q, Zhu L-B, Zhu H, Li T, Li R, Chen S-F, Huang C-P, Zhang X, Zhu J-H (2016). Meta-analysis of brain iron levels of Parkinson’s disease patients determined by postmortem and MRI measurements. Sci Rep.

[6] Pietracupa S, Martin-Bastida A, Piccini P (2017). Iron metabolism and its detection through MRI in parkinsonian disorders: a systematic review. Neurol Sci.

[7] Langkammer C, Pirpamer L, Seiler S, Deistung A, Schweser F, Franthal S, Homayoon N, Katschnig-Winter P, Koegl-Wallner M, Pendl T, Stoegerer EM, Wenzel K, Fazekas F, Ropele S, Reichenbach JR, Schmidt R, Schwingenschuh P (2016). Quantitative susceptibility mapping in Parkinson’s disease. PLoS One.

[8] Du G, Lewis MM, Sica C, He L, Connor JR, Kong L, Mailman RB, Huang X (2018). Distinct progression pattern of susceptibility MRI in the substantia nigra of Parkinson’s patients. Mov Disord.

[9] Uchida Y, Kan H, Sakurai K, Arai N, Kato D, Kawashima S, Ueki Y, Matsukawa N (2019). Voxel-based quantitative susceptibility mapping in Parkinson’s disease with mild cognitive impairment. Mov Disord.

[10] Thomas GEC, Leyland LA, Schrag A-E, Lees AJ, Acosta-Cabronero J, Weil RS (2020). Brain iron deposition is linked with cognitive severity in Parkinson’s disease. J Neurol Neurosurg Psychiatry.

[11] Shin C, Lee S, Lee JY, Rhim JH, Park SW (2018). Non-motor symptom burdens are not associated with iron accumulation in early Parkinson’s disease: a quantitative susceptibility mapping study. J Korean Med Sci.

[12] Postuma RB, Berg D, Stern M, Poewe W, Olanow CW, Oertel W, Obeso J, Marek K, Litvan I, Lang AE, Halliday G, Goetz CG, Gasser T, Dubois B, Chan P, Bloem BR, Adler CH, Deuschl G (2015). MDS clinical diagnostic criteria for Parkinson’s disease. Mov Disord.

[13] Hoehn MM, Yahr MD (1967). Parkinsonism: onset, progression and mortality. Neurology.

[14] Fahn S ER (1987) UPDRS program members. In: Fahn S, Marsden CD, Goldstein M, CalneDB, editors Unified Parkinson’s disease rating scale. Recent developments in Parkinson’s disease vol. 2. p153–163, 293–304

[15] Diederich NJ, Moore CG, Leurgans SE, Chmura TA, Goetz CG (2003). Parkinson disease with old-age onset: a comparative study with subjects with middle-age onset. Arch Neurol.

[16] Yook SKZ (1997). A clinical study on the Korean version of Beck anxiety inventory: comparative study of patient and non-patient. Korean J Clin Psychol.

[17] Choi SH, Na DL, Kwon HM, Yoon SJ, Jeong JH, Ha CK (2000). The Korean version of the neuropsychiatric inventory: a scoring tool for neuropsychiatric disturbance in dementia patients. J Korean Med Sci.

[18] Brown RG, Dittner A, Findley L, Wessely SC (2005). The Parkinson fatigue scale. Parkinsonism Relat Disord.

[19] J S, Baik JYK, Park JH (2005). Parkinson’s disease sleep scale in Korea. J Korean Neurol Assoc.

[20] Frauscher B, Ehrmann L, Zamarian L, Auer F, Mitterling T, Gabelia D, Brandauer E, Delazer M, Poewe W, Hogl B (2012). Validation of the Innsbruck REM sleep behavior disorder inventory. Mov Disord.

[21] Kim J-Y, Song I-U, Koh S-B, Ahn T-B, Kim SJ, Cheon S-M, Cho JW, Kim YJ, Ma H-I, Park M-Y, Baik JS, Lee PH, Chung SJ, Kim J-M, Kim H-J, Sung Y-H, Kwon DY, Lee J-H, Lee J-Y, Kim JS, Yun JY, Kim HJ, Hong JY, Kim M-J, Youn J, Kim JS, Oh ES, Yang H-J, Yoon WT, You S, Kwon K-Y, Park H-E, Lee S-Y, Kim Y, Kim H-T, Kim J-S (2017). Validation of the Korean version of the scale for outcomes in Parkinson’s disease-autonomic. JMD.

[22] Koh SB, Kim JW, Ma HI, Ahn TB, Cho JW, Lee PH, Chung SJ, Kim JS, Kwon DY, Baik JS (2012). Validation of the Korean-version of the nonmotor symptoms scale for Parkinson’s disease. JCN.

[23] LE Lim SY, Jeong SW, Kim HC, Jeong CH, Jeon TY (2011). The validation study of Beck depression scale 2 in Korean version. Anxiety Mood.

[24] Kim JI, Sunwoo MK, Sohn YH, Lee PH, Hong JY (2016). The MMSE and MoCA for screening cognitive impairment in less educated patients with Parkinson’s disease. JMD.

[25] Gudbjartsson H, Patz S (1995). The Rician distribution of noisy MRI data. Magn Reson Med.

[26] Gho SM, Liu C, Li W, Jang U, Kim EY, Hwang D, Kim DH (2014). Susceptibility map-weighted imaging (SMWI) for neuroimaging. Magn Reson Med.

[27] Nioche C, Cabanis EA, Habas C (2009). Functional connectivity of the human red nucleus in the brain resting state at 3T. AJNR.

[28] Colpan ME, Slavin KV (2010). Subthalamic and red nucleus volumes in patients with Parkinson’s disease: Do they change with disease progression?. Parkinsonism Relat Disord.

[29] Enatsu R, Gonzalez-Martinez J, Bulacio J, Kubota Y, Mosher J, Burgess RC, Najm I, Nair DR (2015). Connections of the limbic network: a corticocortical evoked potentials study. Cortex.

[30] Jhe N, Ling H, Ding B, Huang J, Zhang Y, Zhang Z, Liu C, Chen K, Yan F (2015). Region-specific disturbed iron distribution in early idiopathic Parkinson’s disease measured by quantitative susceptibility mapping. Hum Brain Mapp.

[31] Du G, Liu T, Lewis MM, Kong L, Wang Y, Connor J, Mailman RB, Huang X (2016). Quantitative susceptibility mapping of the midbrain in Parkinson’s disease. Mov Disord.

[32] Bergsland N, Zivadinov R, Schweser F, Hagemeier J, Lichter D, Guttuso T (2019). Ventral posterior substantia nigra iron increases over 3 years in Parkinson's disease. Mov Disord.

[33] Aquino D, Contarino V, Albanese A, Minati L, Farina L, Grisoli M, Elia A, Bruzzone MG, Chiapparini L (2014). Substantia nigra in Parkinson’s disease: a multimodal MRI comparison between early and advanced stages of the disease. Neurol Sci.

[34] Liu Z, Shen H-C, Lian T-H, Mao L, Tang S-X, Sun L, Huang X-Y, Guo P, Cao C-J, Yu S-Y, Zuo L-J, Wang X-M, Chen S-D, Chan P, Zhang W (2017). Iron deposition in substantia nigra: abnormal iron metabolism, neuroinflammatory mechanism and clinical relevance. Sci Rep.

[35] Li X, Xing Y, Schwarz ST, Auer DP (2017). Limbic grey matter changes in early Parkinson’s disease. Hum Brain Mapp.

[36] Kalaitzakis ME, Gentleman SM, Pearce RK (2013). Disturbed sleep in Parkinson’s disease: anatomical and pathological correlates. Neuropathol Appl Neurobiol.

[37] Yoo SW, Yun E, Bang M, Yoon U, Yoo JY, Lee KS, Shin NY, Kim JS (2020). Blood pressure lability is associated with subcortical atrophy in early Parkinson’s disease. J Hypertens.

[38] Wang Y, Liu T (2015). Quantitative susceptibility mapping (QSM): Decoding MRI data for a tissue magnetic biomarker. Magn Reson Med.

[39] Rua C, Clarke WT, Driver ID, Mougin O, Morgan AT, Clare S, Francis S, Muir KW, Wise RG, Carpenter TA, Williams GB, Rowe JB, Bowtell R, Rodgers CT (2020). Multi-centre, multi-vendor reproducibility of 7T QSM and R(2)* in the human brain: Results from the UK7T study. Neuroimage.

[40] Lotfipour AK, Wharton S, Schwarz ST, Gontu V, Schäfer A, Peters AM, Bowtell RW, Auer DP, Gowland PA, Bajaj NP (2012). High resolution magnetic susceptibility mapping of the substantia nigra in Parkinson’s disease. J Magn Reson Imaging.

