# Supplementary material for: Extra-basal ganglia iron content and non-motor symptoms in drug-naïve, early Parkinson’s disease
Source: Neurol Sci. 2021 Apr 16;42(12):5297–304. doi: 10.1007/s10072-021-05223-0 (PMC8642382; doi:10.1007/s10072-021-05223-0)
Supplement: Supplementary file 1 — (DOCX 142 kb) [file 10072_2021_5223_MOESM1_ESM.docx]

**Supplementary Material 1.** Scan parameters and settings

|  | T_1_-MPRAGE | Multi-echo gradient echo |
| --- | --- | --- |
| TR | 2000 ms | 47 ms |
| TE | 2.3 ms | 7 to 42 in 5 ms (N_echo_ = 8) |
| Scan duration | 3 min | 8 min 23 sec |
| Field of view | 270(AP) × 189(LR) × 135(SI) | 270(AP) × 186(LR) × 135(SI) |
| Matrix size | 224 × 156 × 112 | 224 × 154 × 112 |
| Voxel size | 1.2 × 1.2 × 1.2 mm^3^ | 1.2 × 1.2 × 1.2 mm^3^ |
| Flip angle | 9° | 20° |
| Pixel bandwidth | 250 Hz | 240 Hz |
| Parallel imaging factor | 2 | 2 |
| Acquisition dimension | 3D | 3D |
| Scan plane | Axial | Axial |
| Flow compensation | N/A | First echo, 3 directions |

*Abbreviations*. MPRAGE, magnetization-prepared rapid acquisition with gradient echo; TR: repetition time; TE: echo time; AP: anterior-posterior; LR: left-right; SI: superior-inferior

**Supplementary Material 2.** Illustration of the process to reconstruct R2* maps


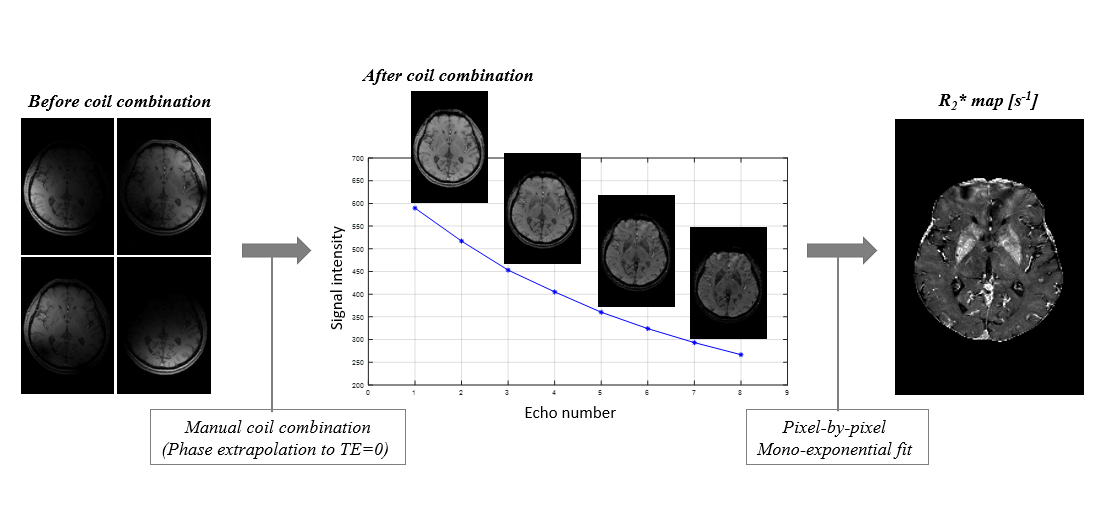


The image data from individual coil channels were processed using an in-house coil combination code. R2* was calculated by pixel-by-pixel mono-exponential fitting of the magnitude data from all echo-times.

**Supplementary Material 3.** Overall variability of R2* and QSM. QSM shows larger within-group standard deviations relative to the inter-group differences compared to R2* (boldface numbers in the last row).

|  | **R2* (s^-1^)** | | | | | | | **QSM (ppb)** | | | | | | |
| --- | --- | --- | --- | --- | --- | --- | --- | --- | --- | --- | --- | --- | --- | --- |
|  | **NC** (n=12) | | **PD** (n=14) | | |  | | **NC** (n=12) | | **PD** (n=14) | | |  | |
|  | mean | stdev | | mean | stdev | | abs(diff) | mean | stdev | | mean | stdev | | abs(diff) |
| **Nucleus accumbens** | 20.7 | 1.9 | | 22.5 | 2.5 | | 1.8 | 5.6 | 9.2 | | 11.8 | 7.5 | | 6.2 |
| **Caudate** | 23.5 | 2.3 | | 25.9 | 3.2 | | 2.4 | 27.8 | 4.5 | | 29.8 | 4.1 | | 2 |
| **Putamen** | 27.8 | 2.9 | | 31.2 | 4.9 | | 3.4 | 25.9 | 7.3 | | 30.3 | 8.6 | | 4.4 |
| **Globus pallidus** | 37.7 | 5.2 | | 39.2 | 4.5 | | 1.5 | 54.9 | 15.1 | | 51.6 | 12.8 | | 3.3 |
| **Thalamus** | 19.8 | 0.9 | | 20.3 | 1.2 | | 0.5 | 0.1 | 2.7 | | -0.5 | 2.6 | | 0.6 |
| **Substantia nigra** | 29.1 | 2.8 | | 31.9 | 3.5 | | 2.8 | 70.3 | 10.6 | | 71.6 | 15.1 | | 1.3 |
| **Red nucleus** | 26.4 | 2.8 | | 28.8 | 3.3 | | 2.4 | 71.2 | 12.3 | | 67.7 | 10.5 | | 3.5 |
| **Dentate nucleus** | 30 | 3.2 | | 31.4 | 2.7 | | 1.4 | 50.2 | 15.8 | | 39.6 | 10.4 | | 10.6 |
| **R. amygdala** | 15.5 | 1.1 | | 16.5 | 1.8 | | 1 | 0.9 | 9.5 | | 0.6 | 7.9 | | 0.3 |
| **L. amygdala** | 16.6 | 1.2 | | 16.5 | 1.5 | | 0.1 | -2 | 7.9 | | 0.7 | 8.2 | | 2.7 |
| **R. hippocampus** | 17.7 | 1 | | 17.7 | 1.6 | | 0 | 1.7 | 4.5 | | 1.9 | 3.2 | | 0.2 |
| **L. hippocampus** | 17.2 | 1 | | 17.4 | 1 | | 0.2 | 0.9 | 2 | | 1.5 | 4.4 | | 0.6 |
| minimum | 15.5000 | 0.9000 | | 16.5000 | 1.0000 | | 0.0000 | -2.00 | 2.00 | | -0.50 | 2.60 | | 0.20 |
| maximum | 37.7000 | 5.2000 | | 39.2000 | 4.9000 | | 3.4000 | 71.20 | 15.80 | | 71.60 | 15.10 | | 10.60 |
| average | 23.5000 | **2.1917** | | 24.9417 | **2.6417** | | **1.4583** | 25.63 | **8.45** | | 25.55 | **7.94** | | **2.98** |

*Abbreviations*. NC, normal control; PD: Parkinson’s disease; stdev, standard deviation; abs(diff), absolute difference between the mean values of NC, PD groups
